# Routes of Well-Being, Spiritual Harmony and Recovery in Mental Health: The Community as a Policy-Maker

**DOI:** 10.34172/ijhpm.8390

**Published:** 2025-02-17

**Authors:** Felipe Agudelo-Hernández, Ana Belén Giraldo-Álvarez

**Affiliations:** ^1^Programa de Medicina, Facultad de Ciencias para la Salud, Universidad de Manizales, Manizales, Colombia.; ^2^Departamento de Medicina, Facultad de Ciencias para la Salud, Universidad de Caldas, Manizales, Colombia.

**Keywords:** Community Health Planning, Community-Based Participatory Research, Culturally Competent Care, Mental Health, Colombia

## Abstract

**Background:**

The participatory approach in policy construction is a historical debt to communities. An example of the above is the definition that Colombian Indigenous communities give to mental health or spiritual harmony. Spiritual harmonies are a collective good that implies being in harmony with the spirit and with thought, which is why it is related to the community context, with the territory, identity, autonomy, spirituality, worldview, diverse cultural uses, practices, and customs. The objective of this study was to analyze the process of building a mental health recovery path with multiple community representatives in a Colombian territory.

**Methods:**

Through a co-production method based on Participatory Action Research, focus groups were developed with representatives of children, youth, indigenous peoples, migrant populations, as well as government sectors such as health, education, and decision-makers in the territory. This study was carried out in 2023 in the District of Riohacha, Colombia, in the last quarter of 2023, within the framework of the construction of a mental health route, led by the District Mayor’s Office. Fifty-nine people participated in the focus groups.

**Results:**

The necessity of living in safe and supportive environments was emphasized. The route built with the community was based on the definition of the necessary steps to generate a real approach to the context and their perceptions of well-being, mental health and spiritual harmonies. Three main themes were identified: (1) Mental health: a construct of well-being, care and identity, (2) Barriers to an integral approach and ideals of joint construction, and (3) The proposal for a comprehensive mental health route.

**Conclusion:**

Co-productive methodologies strengthen community autonomy and empowerment, and make the implementation of mental health programs more feasible. In public policies, it is increasingly necessary to have communities that are strengthened in the production of knowledge and in the proposals for implementation.

## Background

**Key Messages**

**Implications for policy makers**

- It is essential to recognize the meanings that people attach to mental health in order to create roadmaps based on the specific context, resources, needs, and priorities.
- A central barrier to ensuring a comprehensive approach to mental health is that the voice of the communities is not translated into administrative acts, thereby preventing communities from becoming policy-makers.
- It is a priority that, in intersectoral construction spaces, the voice of the communities be recognized as the central factor in the creation and implementation of policies. The term “construction” refers to the process of joint and collaborative creation or co-creation.

**Implications for the public**

This research arises from a process of territorial construction of a public mental health policy, which proposes the route of recovery from mental problems or disorders. It proposes a mechanism to produce public policy guidelines with the communities, to determine barriers and facilitators and propose new ways of implementation. The communities through their representatives, including children, youth, indigenous communities, migrants, and representatives of support groups, show their voice as a central factor in the creation and implementation of policies. This implies recognizing other epistemologies, building knowledge in a transversal way, which dialogues with national and global technical guidelines.

Well-being and mental health constitute pillars for individual and collective development as well recognized in the Sustainable Development Goals.^1^ Thus, improving mental health requires addressing elements such as equity between countries, the living conditions of communities, as well as the identification and contextualized response to the needs of each region. It is evident that the lack of a comprehensive approach to mental health directly impacts individual and social development.^2^

 To illustrate some of the current conditions, the World Health Organization (WHO) estimated for the year 2019 that approximately one billion people had a mental disorder. Suicide represented more than 1% of all causes of death, and mental disorders were the main cause of disability. People with mental disorders face a decreased life expectancy (10 to 20 years), mainly due to preventable non-psychiatric diseases.

 Furthermore, stigma, discrimination and the systematic violation of the human rights of people with mental disorders have been established in the community imagination. The sum of factors generates a reciprocal determinism between adverse living conditions and mental illness, creating a state of mutual feedback.^3^

 Many low- and middle-income countries, such as Latin American countries, face significant disparities that are linked to socioeconomic aspects, geographic location that limits mobilization, barriers to access to social and health services, conditions of violence, as well as identity elements such as ethnicity, gender, age, migration, among others.^4^

 Although extreme poverty has been halved since 1990, which is significant progress, inequities remain deep. Currently, 25% of the population living in developing countries lives on less than $1.25 a day, and many people barely above this threshold remain in a vulnerable situation, constantly exposed to the risk of falling back into poverty.^5^

 Poverty is not only limited to lack of income, but also involves hunger, malnutrition, lack of access to education and basic services, discrimination, social exclusion, and marginalization in decision-making processes. The world’s poorest countries are consistently the most affected by these inequalities, underscoring the urgent need for concrete action to reduce development gaps.^6^

 Against this backdrop, it is crucial that economic growth be inclusive. Development strategies, at both regional and national levels, must be designed and implemented with the active participation of the most affected communities.^6,7^

 The persistence of these gaps is associated with actions conditioned by the availability of resources, the ambiguity of mental health policies, the fragmentation of health systems, and challenges in access to health services.^8^ For this, global public health agendas have redirected their interest towards contextualized community well-being through the development of plans and policies, adopting a participatory and intersectoral approach.^9-11^

 To strengthen community participation, it is necessary to use methods that bring communities closer to the perspectives of other actors and decision-makers.^12,13^ Social medicine and collective health in Latin America offer crucial foundations to understand the social determination of health as a proposal for diagnosing realities.^14^ It also points out a framework to propose changes in health linked to the structural transformation of economic models, with primary care, at the individual and community level, as a foundation.^15^ Using collaborative approaches implies abandoning the perspective of considering people as mere passive recipients of interventions and, instead, recognizing their capacity to influence living conditions.^14^

 Each health program or intervention must be shaped and related in a particular way to the context and the people involved.^13^ Implementation in complex contexts, with multiple levels of interaction, highlights the need to strengthen collaboration between stakeholders in policy construction, program management and research for mental health decision-making.^17^ In the Colombian context, particularly in territories with a large percentage of Indigenous population, and complex situations such as child malnutrition, migration and multidimensional poverty, as is the case of Riohacha, community diversity demands the recognition of these levels of interaction.^18^ However, collaborative approaches are still limited and have low impact on final territorial decisions,^19^ especially in Colombia.^18^

 An example of the above is the definition that Colombian Indigenous communities give to mental health. This is understood as spiritual harmony. Spiritual harmonies are a collective good that implies being in harmony with the spirit and with thought, which is why it is related to the community context, with the territory, identity, autonomy, spirituality, worldview, diverse cultural uses, practices, and customs.^4^

 It is expected that government participation will allow listening and identifying viable mechanisms to respond to the needs raised by the communities.^13^ For their part, representatives of Indigenous and non-Indigenous communities have the responsibility of recognizing the priorities of their communities and proposing viable methodologies that build bridges with other actors.^20^ Establishing regulatory frameworks more aligned with these needs and bringing them effectively closer to communities becomes a task that requires a comprehensive and coordinated approach.^21,22^

 After considering the lack of processes that involve the co-construction of mental health policies in complex implementation contexts, this research aims to analyze the process of building a mental health recovery path with multiple community representatives from the identification of meanings to the proposal of recommendations to sustain well-being, mental health and spiritual harmonies in Riohacha, Colombia. Framed in a participatory methodology, it seeks to recognize people’s perspectives and recommendations for the creation of public policies. On the other hand, Community Based Participatory Research aims to form associations to address community health problems. However, it does not establish dialogue mechanisms between different community levels in the construction of policies. For this reason, it was considered essential to add elements of co-productive methodologies in order to bring together different actors in a purposeful dialogue for the creation of mental health policies.^13^ Transforming communities into policy-makers can facilitate the implementation processes of mental health strategies in Colombia, which have a low implementation at the country level.^18^

## Methods

###  Study Design and Participants

 This research was structured from a qualitative approach, as it sought to observe and understand the subjective reality of the population under study. The qualitative approach allows us to understand the perceptions and experiences of the actors, emphasizing the meaning and significance that they give to their behaviours and their lives within the framework of social interactions. Through a meticulous process of inquiry, qualitative research delves into the specific environment of the participants, both geographically and temporally, to discover and understand the shared meanings and cultural norms that influence their actions and decisions. This approach allows for a deep and nuanced understanding of social dynamics from the perspective of the actors themselves, emphasizing the importance of their context and lived experience.

 This study was carried out in the district of Riohacha, Colombia, in the last quarter of 2023, as part of the construction of a mental health route led by the district mayor’s office. A co-productive methodology was employed to ensure a more horizontal and contextualized research process based on the recognition of autonomy and the history and culture of the community. This method was Participatory Public Health Mapping to actively integrate different sectors and communities in the identification and analysis of mental health needs, resources and barriers in the territory.^23-25^

 Within this approach, “Practical Mapping” was used to collect exploratory data that helped to frame the problem in terms of local interests and experiences.^23^ In addition, elements of the Global Mental Health approach were incorporated to understand the local perception of equity and the guarantee of human rights in their specific context. It was also based on Community Based Rehabilitation to identify the resources and barriers faced by people with health problems and disabilities in their communities, thus facilitating the understanding of their needs based on the recognition of the dynamics of social inclusion and equitable access to resources and services.^25^ Thus, the following steps were developed for its construction (Table 1).

**Table 1:** Steps for Carrying out a Participatory Identification of Needs and Proposals

**Step **	**Description**
Contextualization	Documentary search on public mental health policies in the territory.
Partnerships with the community	Link with community leaders and mapping of actors.
Call for key actors	Invitations to community leaders and the community in general, through social networks, radio and megaphones. To call children and young people, visits were made to the main schools in the territory, including institutions with an ethnic focus.
Research guide built with community leaders	Focus groups to determine barriers, facilitators and recommendations for implementation. The questions were aligned with key themes resulting from the bibliographic search and conversations with community representatives.
Construction of the route	The interviews were recorded with the consent of the participants. The minutes were shared with the groups, who were able to make contributions to them.
Presentation of the route	After the mental health route was built with the contributions and review of community leaders and actors in the health system, a document was created that was signed by the mayor of the Riohacha District, through an administrative act to recognize the route of well-being and recovery.

 To this end, the following trigger questions were proposed: What is mental health for you? What is mental health like in Riohacha? What barriers or difficulties do people in your community have to sustain or recover mental health? How do you imagine an effective recovery process for yourself or someone close to you? Who would you have to turn to, what services would you go to? What institutions or sectors, and under what circumstances, should intervene to address mental health?

 Each group was attended by an intercultural leader, a social worker from the Wayuú People, a researcher accepted by the focus groups (psychiatrist, Ph.D in social sciences) and a researcher with no previous contact with the participants (psychologist, Ph.D in psychology). The triggering questions initially allowed for nurturing the debate around the understanding and delimitation of the main problems in relation to mental health. From this, a space for brainstorming was generated which, according to the possibilities and resources of the environment, were directed towards more precise plans that finally constituted one of the fundamental inputs for the District.

 After the discussion around the questions, the group of mental health experts who were in the territory took as a basis the needs and proposals of all the participants in order to establish the key issues for the construction of the route. A comprehensive review of strategies, tools and programmes at regional, national and global levels, as well as of health and mental health regulations, was carried out, based on the central components identified in the focus groups and their corresponding subtopics. This analysis was aimed at promoting the implementation of a comprehensive mental health route, taking into account local needs and resources.

 The integration process included combining the analysis derived from the focus group discussions with the strategies and methodologies proposed at global, national and regional levels in the field of mental health, in addition to the regulations in force in this field. The result was the formulation of a broad and comprehensive route that addresses population differences, considering specific vulnerabilities, ethnicity, gender diversity, exposure to violence and life course in a contextualized manner. After the construction of the route, the final versions were jointly reviewed in the territory in order to evaluate the product that constitutes the action route. Subsequently, this information was used to create the Riohacha Mental Health Route and its implementation milestones. This document was signed by the mayor of the District in December 2023 to integrate it into the territory’s health regulations.^26^

###  Analysis of Data

 The systematization and analysis of the data was done simultaneously with the field work. This favoured making adjustments to the techniques, delving deeper into topics, and investigating in greater depth for emerging themes.^27^ The field work was carried out until data saturation was reached. In the context of qualitative research, this means that information continued to be collected until no new themes, patterns, or insights emerged in the data. Saturation is an indicator that the phenomenon under study has been thoroughly explored and that enough information has been collected to fully understand it. It is a point at which adding more data does not add additional value or offer new perspectives on the topic being investigated.

 Achieving saturation is essential to guarantee the validity and depth of the study, ensuring that all facets and dimensions of the phenomenon in question have been considered. In addition, this approach avoids unnecessary data collection, optimizing resources and time. For the evaluation of the data collected, an approach focused on content analysis was chosen. Initially, the interviews and focus groups were transcribed verbatim. Subsequently, the data were coded and categorized using ATLAS.ti software, version 23, grouping the categories according to their intrinsic characteristics and content. During the structuring phase of the analysis, an endogenous interpretation perspective was adopted, based on the internal analysis of the data, as well as on the previously defined categories and those that emerged during the study, which were integrated with relevant theories and findings from previous research.

 In order to ensure the validity of the results obtained in the research, a triangulation process was implemented. This process involved comparing and contrasting the data collected through different techniques, participants and groups involved in the study. Additionally, the project design, the system of categories used and the progress achieved in the field were subject to evaluation by experts in the field. This step was carried out with the aim of enriching the perspective of the study and improving the applied research methodology. The discussion on the progress made and the results obtained was also extended to the participants of the groups involved, recognizing that the legitimacy of the knowledge acquired is strengthened through shared experiences and consensus reached through dialogue.

 Although no Indigenous reviewers were directly involved, a consensus on the topics was achieved through an inclusive and collaborative approach. Extensive dialogues with Indigenous communities were conducted during the study phase to identify their needs and perspectives, adapting the design and analysis of the information accordingly. Previous experience working with these communities provided important historical and cultural context, allowing for a better understanding of the unique mental health vulnerabilities of these populations. While direct Indigenous participation in the review was not possible, significant efforts were made to integrate their perspectives and ensure that the study accurately reflected the cultural and social context.

## Results

 A total of 59 people participated in the focus groups, with a mean age of 38 and a median age of 40. Of these participants, 41 were women, representing 69.5% of the group. Representatives of children, adolescents, young people, the ethnic sector, migrants, the educational sector, and the health sector participated. Representatives from other sectors also participated, such as businessmen, media, justice sector, international cooperation, and community leaders (Table 2).

**Table 2:** Participants in the Focus Groups

**Group Represented**	**Average Age**	**Women**	**Men**	**Occupation/Profession**
Indigenous (I)	44	3	6	Psychologist (2), housewife (1), farmer (2), teacher (2), various trades (2)
Health secretary (HS)	45	2	1	Psychologist (2), nurse (1)
Education sector (ES)	46.8	3	3	No Indigenous teacher (6)
Afro-descendant (A)	59	0	2	Psychologist (1), housewife (1)
Edil* (E)	46	1	1	Teacher (1), various trades (1)
Mayor (M)	53	1	0	Lawyer (1)
Childhood and youth (CY)	12.6	7	4	Student (11)
Support group leader (GS)	48	4	2	Housewife (3), occupational health technician (1), university student (2)
Media (M)	56	0	1	Businessman (1)
Penal system (P)	42	0	1	Social worker (1)
Migrant population (MP)	40.5	2	2	Various trades (2), unemployed (2)
International cooperation (IC)	37.2	5	1	Social worker (3), psychologist (2), nurse (1)
Health benefit plan administrative entity (HB)	35.8	8	1	Social worker (2), psychologist (4), nurse (3)

* Community leader elected by popular vote.

 The results that emerged in the analysis were guided by the definition of mental health as spiritual harmony. The route built with the community was based on the definition of the necessary steps to generate a real approach to the context and their perceptions of well-being, mental health and spiritual harmonies. The research methodology was established according to the indications of the community.

 In relation to the analysis and organization of the information, three main themes were identified: (1) Mental health: a construct of well-being, care and identity, (2) Barriers to an integral approach and ideals of joint construction, and (3) The proposal for a comprehensive mental health route. From these themes, sub-themes were defined that allow for a more detailed description of their components. Next, the thematic analysis resulting from the focus group dissertation is presented and emerging themes and related recommendations are proposed. The citations are provided according to the group represented they belong to and the assignment number given by the analysis program coding. The above, to preserve confidentiality.

###  Mental Health: A Construct of Well-Being, Care, and Identity

 The different groups stated that mental health is a process that involves being able to grow, work, play, dream, live, and relate in a safe environment on an individual and collective level. Thus, representatives of children and adolescents began by recognizing: *“Having a place for yourself where you can be yourself”* [CY_1]. To this, one of the participants, on behalf of another group of children and adolescents responded: *“I drew a flower, because of how mental health is born […] Mental health is something that we have to take care of so that it does not wither”* [CY_2].

 Mental health was illustrated as a process that requires patience and permanent construction. Furthermore, they recognized that it is a dynamic process with a spectrum of emotions and situations: *“That cloud represents the person and the different situations that a person may be in, because people go through different situations that make us feel different ways, and that is also what mental health is about.”* [CY_3]. From the Educational Sector they complemented the characterization of mental health through drawings, transferring their understanding to children, young people and the community:

 “*I will describe it through a house, which has a roof, reflecting security and can also be represented in a school, in a health center, because what guarantees emotional balance for me? The house, because it is a protective environment”* [ES_1].

 Another member added: *“We must also take into account the social environment in which we are developing, since we are biopsychosocial beings, therefore we need to have good interpersonal relationships […]”* [GS_2]. They also recognized the importance of mutual support:

 “*All united, represented as a tree, where independent of any social condition, any economic, cultural, political condition, that tree means the support that in one way or another we all need on that long path that we have to walk”* [GS_5].

 Members of the Indigenous Communities stated that a focus on spiritual harmonies is required, understanding people as integral beings: *“Mental health is a balance that can be achieved with the harmonization of our spiritual processes, that means the realization of the soul, body and spirit and also thought […]”* [I_3]. And it is added: *“The word worldview within Indigenous peoples is important, because it is simply how we, Indigenous peoples, see mental health […]*” [I_5].

 The representatives of Community Leaders recognized the clear links between mental health and the guarantee of other decent living conditions: *“The lack of mental health is an imbalance in our home due to lack of opportunities, […] as councillors and community leaders we must be prepared to even provide relief or know how to act to help our community”* [E_2]. Also, from the representatives of support groups for mental health, it was mentioned:

 “*There are people who get up thinking where they are going to get that day to day to be able to feed themselves, to be able to seek their well-being, that leads to the person getting up with emotional stress”* [GS_6].

 From the District Health Secretariat, they added that it is a process that transcends individual well-being, and they exalt the impact of the pandemic. *“Mental health changes according to the situations you are experiencing and the conditions in which these situations occur”* [M_3]. It was complemented: *“As a result and after the pandemic, we have noticed that mental health problems and illnesses are increasing, let’s say that a new pandemic has occurred, mental problems and disorders”* [P_2].

 They also reinforced the importance of the support and commitment of families in the recovery processes. The lack of family support and solid support networks is recognized as an obstacle to improving mental health:

 “*The family as that nucleus of society that guides them, instead, then puts barriers in their access to the service […]. There is no support from family members and there should be a good support network, because although the doctors, the health area or the representatives of the Ministry of Health are attentive, it is not enough”* [MP_4].

 In this regard, another of the participants added, regarding violence: *“I also think that domestic violence is another factor, another weakness within homes […]”* [IC_2].

###  Barriers to an Integral Approach and Ideals of Joint Construction

 The group of leaders recognized that there are not enough opportunities and highlighted the importance of guaranteeing decent living conditions such as work, education and recreation: *“We as social leaders who are here, notice that what we really need is entertainment and dignified work for each person who lives in our community*” [GS_3]. This comment was complemented by another leader:

 “*The lack of opportunities and not having a decent job is also a barrier that we have, if we look at 10 households there are three that are working and the rest that are not. Our young people also lack educational opportunities […] there are young people who enrol and sometimes there are refusals, who do not pass the process; In education we have to pay a little more attention, because our young people are not being educated, in the pandemic there were many children in the district who lost the year, because they did not even have a cell phone to take a class”* [GS_7].

 Another member added that greater government and other support is required to improve housing and recreation spaces:

 “*Housing programs for vulnerable people, who can acquire decent housing and their needs are mitigated. Our commune lacks a sports center, that is what we want and need”* [HS_3].

 For their part, Indigenous peoples comment that a fundamental barrier for them is the lack of an intercultural approach, they report that: *“There is little knowledge among health professionals about the traditions, uses, customs and traditional practices of Indigenous peoples”* [I_5]. They also highlighted the need to bring ethnic and Western approaches closer together: *“Lack of articulation of the processes of traditional medicine and Western medicine to guarantee comprehensive care with a differential focus on the mental health of the population”* [I_4].

 Likewise, the need for regulatory elements that integrate traditional forms of recovery with Western ones was pointed out: *“There is a need for an approach in psychology that can bring together native procedures of the Wayúu people and the Wiwa, and seek to integrate with the procedure carried out by the psychologist”* [I_2]. The lack of mental health education was added as a barrier: *“Little psychoeducation based on the conception of individual and collective life, where ancestral wisdom is essential to guide mental health”* [I_5].

 Migrant Representatives also highlight gaps in psychoeducation: *“Little psychoeducation based on the conception of individual and collective life, where ancestral wisdom is essential to guide mental health. There is little training in mental health with an intercultural approach […]”* [MP_5]. And they also emphasized the importance of the educational sector getting involved in psychoeducation:

 “*And in the educational system we have to work on emotional intelligence, the management of emotions, which is not the same as disguising or pretending happiness that does not exist. And to achieve this, the approach has to be comprehensive, multifactorial, that is, contextualized. Emotional education is not the same for people who live in a city as it is for people who live in an Indigenous community. Even the language must be adapted depending on the people and the one that one is going to address”* [MP_2].

###  Proposal for a Comprehensive Mental Health Route

 Here some factors not previously mentioned were added and emphasis was placed on others for the construction of the route. The members of the Migrant Group and Afro-descendant raised intersectoral work as a priority:

 “*I think this is how the route would be: Community, Family, health administrators, and the monitoring would be done by the territorial health entities, both territorial and family, and other actors to continue with the treatment, whether pharmacological or otherwise, in the community, which is where real life happens”* [MP_7].

 Understanding mental health from a broad approach was noted as a necessity for the creation of the route: *“Here in care and rehabilitation, mental health must always be a priority, even the legal part must be there because there are people who are victims of psychological violence and have effects on their mental health”* [A_2].

 They highlighted the importance of support groups as a component in the entire approach process, integrated with care and education: *“Support group for prevention and education, emotional education, education and health in a comprehensive and multifactorial manner, contextualized to the reality of the individual and everything has to be group and individual”* [MP_4]. In addition, they added the importance of having community leaders who support mental health: *“A community leader is essential, because we know that, if we approach from the community, from the house and even a neighbour, we can route this person so that he can receive help” *[A_4].

 The Justice Sector considers it important to have adequate tools to provide initial mental health support: *“That they train all of us, all the human talent so that we are prepared to address mental health in these cases […]”* [P_2]. The district health office emphasizes the importance of working to reduce stigma: *“The family must be educated in the stigma reduction of mental health, in the recognition of mental health problems and in the ways to act against them”* [HS_4]. In addition, they mentioned regarding the participation of peers: *“Peers are essential in the mental health and well-being of children and young people; friends can more easily recognize the suffering of others”* [HS_5].

 The role given to family and social support is a factor that is highlighted as a determining factor in mental health. A leader of the adolescents noted: *“Sometimes you have to rely on your family, first, or with that friend you support yourself psychologically, socially and then address all the psychologist’s instructions”* [CY_7].

 The strategies that they considered fundamental for their mental health were discussed with the Indigenous communities and it was found that the Unique Classification Codes for Health Procedures (codes so that health procedures can be recognized and paid for) did not reflect, as part of prioritized activities, traditional practices. This is how the group of Indigenous leaders proposed the translation of the Unique Classification Codes for Health Procedures codes into Traditional Actions for Mental Health with an intercultural approach. This translation was designed with the Indigenous leaders and was socialized and discussed with the Indigenous communities, resulting in the information shown in Table 3.

**Table 3:** Traditional Actions for Recovery Equivalent to the Colombian Health System

**Western Services in the Colombia Health Benefit Plan**	**Traditional Actions for Mental Health With an Intercultural Approach**			
	**Prevention Actions in Mental Illnesses**	**Mental Healthcare Actions**	**Traditional Treatment**	**Interdisciplinary Team**
Intervention in community mental health, through psychiatry and general medicine	Positive thoughts session for the pregnant mother	Comprehensive care with an intercultural approach	Therapy focused on harmonization of the soul, spirit and thoughts	Mamo*, Saga*, Osu*, psychiatrist, psychologist, intercultural psychologist, social worker, general practitioner
Intervention in community mental health, by nursing	I confess in thoughts of mother and father	Sheurruama* approach application	Spiritual confession	
Intervention in community mental health, through social work	Spiritual cleansing mother and father	Attention on the hill – sacred site	Spiritual cleansing	
Intervention in community mental health, through occupational therapy	Newborn baptism	Clinics with an intercultural approach	Spiritual healing	
Intervention in community mental health, by another health professional	Newborn “Gon”	Hospitals with an intercultural approach	Spiritual payment	
Mental health interventions in the community	Young or adult gayama	Mental units with an intercultural approach	Traditional negative soul therapy	
Individual psychotherapy	Intercultural psychoeducation	Articulation of traditional medicine and professional techniques	Traditional positive soul therapy	
Crisis intervention Couples’ psychotherapy			Traditional negative spiritual therapy	
Family psychotherapy Group psychotherapy			Traditional positive thinking therapy	

* Ancestral Knowledgest.

 Other contributions from sectors such as international cooperation, health administration entities, the business sector, support groups and the first level of healthcare are shown in Table 4 as emerging subthemes and recommendations resulting from the deliberation.

**Table 4:** Central Components and Subthemes, Focus Groups

**Main Themes/Categories**	**Specific Subtopics/Recommendations for Route Construction**
Meanings of mental health or spiritual harmony	*Spiritual and mental harmony* • Seek a spiritual balance with thoughts, emotions and actions. • Support and healing from Indigenous knowledge. *Care and protective environment* • Have a safe environment where permanent and sensitive care is taken. *Supportive and understanding environment* • Promote support, accompaniment, understanding, active listening, and empathetic communication in different spaces of life, mainly at home, in the community and at school. *Mental health and wellness education* • Adapt education to the needs of the community, the educational sector, the labor sector, the government sector, and other cooperating institutions. *Basic needs guarantee* • Have coverage of fundamental needs such as housing, services, food, education, and employment. *Influence of the environment* • Recognize the impact on mental health of the environment, including factors such as coexistence, living space and the environment and link them in understanding and addressing mental health. *Importance of recreational spaces* • Recognize recreational, sports and cultural spaces as settings to maintain mental health. *Development of capabilities and opportunities* • Enable the development and strengthening of skills in an environment that provides opportunities.
Barriers	*Barriers to care* • Difficulty accessing mental health resources and professionals. • Socioeconomic barriers that affect adequate care. • Delays on the part of health and administrative institutions to provide mental healthcare. • Lack of implementation of early attention services. *Stigma, fear and shame* • Public stigma and discrimination. • Self-stigma that prevents the search for support. *Lack of intersectorality* • Lack of coordination between the educational and health sectors, mainly. • Difficulties in communication between different entities. • Presence of limited and isolated actions. *Lack of knowledge* • Lack of mental health education. • Lack of knowledge of mental health programs and routes. • Lack of knowledge of the existence of support groups or other social resources. *Socioeconomic factors* • Economic conditions that prevent the guarantee of basic conditions of well-being and that hinder the maintenance of mental health or the seeking of care. • Unemployment and lack of job opportunities. *Cultural challenges* • Little contextualized approaches. • Difficulties in communication with Indigenous communities. • Little knowledge of cultural traditions in Western care.
Recommendations	*Improvement in mental healthcare* • Strengthening mental health resources and services. • Reduction of socioeconomic barriers for more equitable care. • Improvement of continuing professional education processes in mental health for low complexity levels of care. • Establishment of continuous mental health monitoring processes. • Implementation of early attention services. *Integration of the approach of Indigenous communities with the Western approach* • Coordination of the worldview of Indigenous peoples with Western practices. • Intersectoral integration for the recognition of different needs. *Stigma reduction* • Contextualized and culturally appropriate stigma reduction educational programs. • Promoting safe and supportive environments for seeking help. *Intersectoral Coordination* • Designation and strengthening of meeting spaces between the different sectors where responsibilities are agreed. • Improvement in coordination between the educational and health sectors, making use of other social and institutional resources that are required. • Establishment of clear communication protocols between entities. *Education and awareness* • Establishment of educational programs on mental health at the community level differentiated by age groups, with special emphasis on the education of families as adequate care, upbringing and support environments. • Mental health awareness and education in educational institutions and workplaces. *Family and community support* • Strengthening the family and community support network through the promotion of support groups. • Involvement of community leaders in the entire promotion, prevention, care, and monitoring process. *Culturally appropriate and sensitive interventions* • Development of interventions contextualized and sensitized to cultural diversity. • Training of health professionals in cultural competence. *Strengthening working and educational conditions* • Coordination of employment and educational opportunities. • Linking work environments in mental healthcare strategies. *Holistic approach and public mental health* • Development of broad and comprehensive approaches that address mental health from its foundations. • Care programs that promote emotional well-being from an early age in all environments.

## Discussion

 This study sought to analyze the construction of a mental health route from multiple community representatives, in dialogue with policy-makers. This process was based on the adaptation of collaborative approaches in the methodological framework of Participatory Action Research for the construction of public mental health policies in the District of Riohacha, a multicultural, multiethnic municipality with complex scenarios for the implementation of health.^17,18,21^

 This change in focus allows the strengthening of community services and primary care, which are based in the territories themselves.^28^ For this reason, participatory methodologies have been proposed that seek to effectively incorporate the visions of different actors in a balanced and intercultural manner.^29,30^

 Mathias et al^29^ sought to explore the nature of community mental health systems through a participatory community assessment of mental health resources and needs in a district in northern India and found that the real needs of communities can be better understood through participatory methodologies, where gaps were identified in the hierarchies of approach, system fragmentation and quality of services, so that the community recommended a real rapprochement between community and public health systems that respond to the context, their needs and resources. These findings are similar to those of our study in terms of the elements that the population highlights in relation to the fact of perceiving fragmentation among the different actors and spaces.^29^

 The meanings given to mental health also guide the identification of needs, which was highlighted in this research from the construction of well-being, mental health and harmonies with others, at the family and community level and with the participation of different sectors. In this sense, the establishment of intersectoral links from the beginning of the interventions has been proposed as a fundamental factor.^30^

 This gap has also been recognized in patient organizations where not being listened to represents a central factor in the persistence of problems.^31^ The meanings given to mental health also guide the identification of needs, which was highlighted in this research from the construction of well-being, mental health and harmonies with others, at the family and community level and with the participation of different sectors. In this sense, the establishment of intersectoral links from the beginning of the interventions has been proposed as a fundamental factor.^32-34^ Gaps in participation have represented a cross-cutting issue in the barriers identified worldwide when reviewing mental health conditions. In the qualitative metasynthesis “Participation in mental healthcare” conducted by Stomski and Morrison,^33^ they found that community participation that translates into public policies is scarce and that change must be generated by the community itself.

 In Colombia, co-constructive efforts in health are still incipient since there is a lack of effective linkage that addresses community needs^18^. Furthermore, citizen participation has not had a real influence on decision-making.^35^ Gil et al^36^ described community social participation as a strategy for the construction of a comprehensive health management model with rural populations in the Colombian Pacific and concluded that community participation is crucial for the development of comprehensive health models in these areas.

 In addition to barriers to participation, intersectionality has faced significant challenges. Among these, the scant consideration of community perspectives in the formulation of final strategies discourages community participation in these spaces.^36,37^ For this reason, this study started from dialogue with and between the different actors in the territory in order to recognize shared responsibilities in the development of actions.

 Regarding the identification of central topics in mental health and the generation of recommendations, it is observed that many of the essential elements align with topics addressed at a global level in mental health.^37,38^ The marked disparities in access to health services, in addition to the challenges in the continuity of care and in the contextualization of mental health approaches are issues that must be addressed along the route.

 The generation and strengthening of support and care networks is a fundamental factor for individual and community well-being. When it occurs in the context of support or mutual aid groups, it generates greater chances of transcendence for people with a psychiatric diagnosis, in addition to allowing capabilities to be enhanced.^21,39^ Healthy, supportive and caring family, school and work environments have also been essential factors for maintaining mental health and strengthening rehabilitation processes at the global and local levels. Furthermore, the guarantee of opportunities and decent living conditions are basic factors to enhance community well-being.^40^

 Intersectorality continues to be a task that cannot be postponed for a comprehensive approach and to provide effective responses to different needs, with special emphasis on the discussion of disparities in financing in global mental health and the allocation of resources, since the majority of these resources continues to be assigned to psychiatric clinics, despite worldwide recommendations that point to the opposite.^41^ Intersectorality would also allow us to face the challenges of implementation.^21,42^ The need for intercultural approaches adapted to contexts is a national commitment that must be put into practice locally.^4^

 From the characterization of community perceptions and recommendations, it is expected to build a mental health path that includes the emerging themes of this study. It is essential to review and reform existing regulatory frameworks to ensure that they more closely reflect the realities and demands of communities. Adapting policies and regulations to the specific needs of each region can contribute significantly to closing gaps in access and improving the quality of health services.^43^

 Therefore, taking into account the difficulties to be heard and to really participate, using a collaborative methodology allowed listening to their voices and recognizing what they would expect to find in a path of mental health, well-being and spiritual harmonies. Based on the recognition of barriers, recommendations and the imaginary of a pathway, it was found that the community recognizes the value of the clinical care actions that are available, but these must be framed in the context and based on actions that involve different people in their community roles and in the family.

 The developed route proposes establishing points of contact between different stakeholders, integrating clinical actions with community and cultural elements. In this context, a community-based approach to mental health offers a higher likelihood that individuals will follow the steps they have outlined themselves and maintain a process of monitoring, oversight, and dynamic adjustment that responds to their own needs, priorities, and resources, from a perspective of greater autonomy and empowerment.

###  Limitations 

 Although having an administrative act that makes the voice of the communities normative is recognized as a strength, the absence of costing to make the implementation of these recommendations more feasible is stated as limitations. Likewise, the absence of other sectors necessary for recovery is noted. Future studies could build implementation plans for these recommendations.

 Likewise, it is acknowledged as a limitation that there were no reviewers from Indigenous communities in the methodological process and in writing the manuscript. Although non-Indigenous reviewers did their best to the extent possible to use their experience within the framework of cultural sensitivity and humility, there is no replacement for Indigenous review and content may have been omitted if that had been possible. This invites future studies to better empower Indigenous communities before starting a research process, to reduce the risk of producing knowledge without equity.^44^ In this case, that was not possible because the development of this research took place in the construction of a mental health route led by a government organization.

## Conclusion

 The results reinforce the concept of mental health as spiritual harmony, which mentions the integration of the community, the family, the territorial, and the individual. When community members with lived experience from intersectoral groups are engaged more authentic knowledge that has high potential to produce and inform frameworks that will make a higher impact on reducing mental health.

 This process has shown that the construction of guidelines is not enough to impact collective well-being. Communities, through their representatives, including children, youth, indigenous communities, migrants, and representatives of support groups, show their voice as a central factor in the creation and implementation of policies. This implies recognizing other epistemologies in the construction of situated knowledge that dialogues with national and global technical guidelines.

## Acknowledgements

 To Yair Villazón Mindiola for his constant accompaniment and care in the methodology of this process. To Wiwa and Wayuú Peoples.

## Ethical issues

 This study complies with the research ethics guidelines for human subjects as outlined in Resolution No. 008430 of 1993 by the Ministry of Health and the Helsinki Declaration of 2000. It is a minimal-risk research study and it was reviewed and approved through the CBE02_2022 resolution by by Ethics Committee University of Manizales. All participants signed an informed consent or assent to voluntarily accept the recordings, the construction of the route and the study in a culturally appropriate manner, ensuring confidentiality through data anonymisation and secure storage. Traditional knowledge was protected through agreements with community leaders on respect for cultural rights. During the process, psychosocial support was offered by trained mental health professionals and a process of follow-up and ongoing support for participants was agreed. In addition, community leaders were involved in reviewing the research process and staff were trained in cultural recognition and sensitivity to ensure respectful and safe research.

## Conflicts of interest

 Authors declare that they have no conflicts of interest.
